# RBC Membrane Camouflaged Semiconducting Polymer Nanoparticles for Near-Infrared Photoacoustic Imaging and Photothermal Therapy

**DOI:** 10.1007/s40820-020-00429-x

**Published:** 2020-04-20

**Authors:** Dongye Zheng, Peiwen Yu, Zuwu Wei, Cheng Zhong, Ming Wu, Xiaolong Liu

**Affiliations:** 1 School of Life Sciences, Fujian Agriculture and Forestry University, Fuzhou, 350002 People’s Republic of China; 2 The United Innovation of Mengchao Hepatobiliary Technology Key Laboratory of Fujian Province, Mengchao Hepatobiliary Hospital of Fujian Medical University, Fuzhou, 350025 People’s Republic of China; 3 Key Laboratory of Design and Assembly of Functional Nanostructures, Fujian Institute of Research on the Structure of Matter, Chinese Academy of Sciences, Fuzhou, 350002 People’s Republic of China; 4 Mengchao Med-X Center, Fuzhou University, Fuzhou, 350116 People’s Republic of China; 5 Department of Chemistry, Hubei Key Lab on Organic and Polymeric Optoelectronic Materials, Wuhan University, Wuhan, 430072 People’s Republic of China

**Keywords:** Semiconducting conjugated polymer nanoparticles, Red blood cell membrane camouflage, Deep tumor penetration, Photoacoustic imaging, Photothermal therapy

## Abstract

Semiconducting conjugated polymer nanoparticles (SPNs) represent an emerging class of phototheranostic materials with great promise for cancer treatment. In this report, low-bandgap electron donor–acceptor (D–A)-conjugated SPNs with surface cloaked by red blood cell membrane (RBCM) are developed for highly effective photoacoustic imaging and photothermal therapy. The resulting RBCM-coated SPN (SPN@RBCM) displays remarkable near-infrared light absorption and good photostability, as well as high photothermal conversion efficiency for photoacoustic imaging and photothermal therapy. Particularly, due to the small size (< 5 nm), SPN@RBCM has the advantages of deep tumor penetration and rapid clearance from the body with no appreciable toxicity. The RBCM endows the SPNs with prolonged systematic circulation time, less reticuloendothelial system uptake and reduced immune-recognition, hence improving tumor accumulation after intravenous injection, which provides strong photoacoustic signals and exerts excellent photothermal therapeutic effects. Thus, this work provides a valuable paradigm for safe and highly efficient tumor photoacoustic imaging and photothermal therapy for further clinical translation.

**Electronic supplementary material:**

The online version of this article (10.1007/s40820-020-00429-x) contains supplementary material, which is available to authorized users.

## Highlights

- A narrow bandgap electron donor–acceptor (D–A) semiconducting polymer nanoparticle (SPN) coated with red blood cell membrane (RBCM) for photoacoustic imaging and photothermal therapy.
- The D–A structure endows SPN with excellent near-infrared absorbance, high photothermal conversion ability, and good photothermal stability.
- The RBCM endows SPN with good biocompatibility, prolonged blood circulation, and improved tumor accumulation, while the small size structure endows SPN with deep tumor penetration and rapid clearance from body.

**Electronic supplementary material:**

The online version of this article (10.1007/s40820-020-00429-x) contains supplementary material, which is available to authorized users.

## Graphical abstract

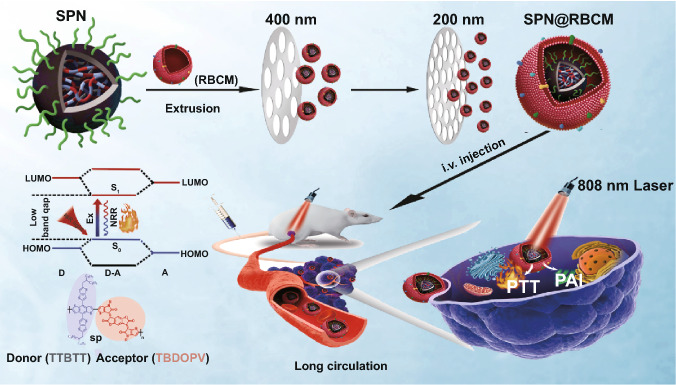

## Introduction

Theranostic nanoplatforms simultaneously bearing diagnostic and therapeutic functions through a single entity are crucial for detecting diseases at an early stage and realizing image-guided therapies, which is favored to improve the survival rate of cancer patients [1, 2]. Recently, phototheranostic nanoagents utilizing light to collect diagnostic information and achieve therapeutic effects have gained intensive attention due to their noninvasiveness, high efficiency, and convenient maneuverability in a spatial and temporal manner [3, 4]. Among the numerous phototheranostic strategies, photoacoustic imaging (PAI) and photothermal therapy (PTT) can seamlessly and synergistically match each other, because the efficacies of both PAI and PTT are mainly dependent on photothermal conversion ability, considering that PAI uses photothermally converted acoustic waves to realize ultrasound signal detection [5–8]. PAI can overcome the traditional optical penetration limitations (e.g., fluorescence imaging) since it detects phonons rather than photons under light excitation, providing superior spatial resolution, high contrast, and deep tissue penetration [7, 9–11]. To date, various optical agents have been developed for PAI/PTT theranostics, including small molecule dyes, metallic nanoparticles, and carbon nanomaterials [12–16]. Nevertheless, small molecule dyes usually suffer from photobleaching, while metallic nanoparticles encounter severe biotoxicity and carbon nanomaterials have broad PAI spectra without a specific wavelength and a low extinction coefficient for PAI and PTT [17, 18]. Semiconducting polymer nanoparticles (SPNs) characterized by *π*–π electron delocalized backbones and easy exciton diffusion along the backbone represent a new PAI/PTT agent with good photostability, high extinction coefficient, and excellent photothermal conversion efficiency [1, 5]. As SPNs are fabricated from biologically inert organic compounds, they can inherently avoid of heavy metal ion-induced toxicity to tissues and thus possess good biocompatibility [19]. However, narrowing their absorption bandgap within the near-infrared light (NIR) window along with a high extinction coefficient is challenging but essentially needed, which may facilitate efficient NIR photothermal conversion for PAI and PTT [20]. In addition, similar to other nano-scaled biomaterials, artificial phototheranostic nanoagents generally encounter several obstacles in clinical translation, including easy recognition by the immune system, insufficient accumulation in the tumor site, and safety concerns regarding physiological interactions and metabolism, which compromise their potential for clinical practice [21, 22].

Surface decoration of phototheranostic nanoagents with synthetic polymers or targeting ligands is an efficient approach to mitigate these issues, but the complicated process as well as the possibility of activating the immune system might further hinder their applications [23, 24]. Biological membranes have been exploited to camouflage nanoparticles for optimized cancer theranostics though reducing side effects, prolonging circulation time, and improving targeting ability [25–28]. Many kinds of cell membranes have been utilized to construct biomimetic nanoparticles. Such a strategy has become extremely attractive as the surface biomolecules inherited from the sourced cell membranes being capable of rendering biomimetic particles with special biological functions. For example, erythrocyte membrane coating helps various nanoparticles suppress immune attack due to the presence of “self-markers” (e.g., the CD47 protein). Indeed, red blood cell membranes have been used to cloak poly(lactic-co-glycolic acid) [25], magnetic [29], perfluorocarbon [30], gold [31], metal organic framework (MOF) [32], and drug-based crystal [33] nanoparticles for cancer treatment. In addition, this top-down method extensively simplifies the procedure for the surface modification procedure of nanoparticles [34]. However, relevant reports adopting this approach for constructing biomimetic SPN-based phototheranostic agents are still very rare but highly desirable. Very recently, Pu and co-workers introduced a biomimetic SPN-based nanoparticle (AF-SPN) coated with the cell membranes of activated fibroblasts for the enhanced cancer phototheranostics [35].

Although improved theranostic efficacy has been achieved by a cell membrane camouflaging strategy, other bottlenecks still exist that cause the poor performance of nanoparticles in solid tumor treatment. It has been realized that nano-based therapeutics mainly deliver particles to the cells on the tumor periphery, owing to the dense interstitial structure of the tumor that impedes the penetration of external nanoparticles to reach a therapeutic dose [36–38]. Moreover, to avoid potential long-term toxicity, the theranostic agents should be biodegradable or clearable within an appropriate period for clinical applications. In particular, the size of nanoparticle has a great influence on the toxicity and clearance characteristics [39–41]. For example, small-sized nanoparticles (less than 30 nm) have been proven to exhibit deep tumor penetration [42–44]. Meanwhile, it is also well known that ultrasmall-sized nanoparticles possess renal clearance characteristics, such as gold nanoparticles, black phosphorus quantum dots, porphyrin-PEG polymers, and metal/covalent-organic framework nanodots [39, 41, 45–47]. Thus, elaborately designed nanoparticles with a reasonable size and appropriate surface decoration can balance the demands between body clearance and tumor accumulation.

**Scheme 1:**
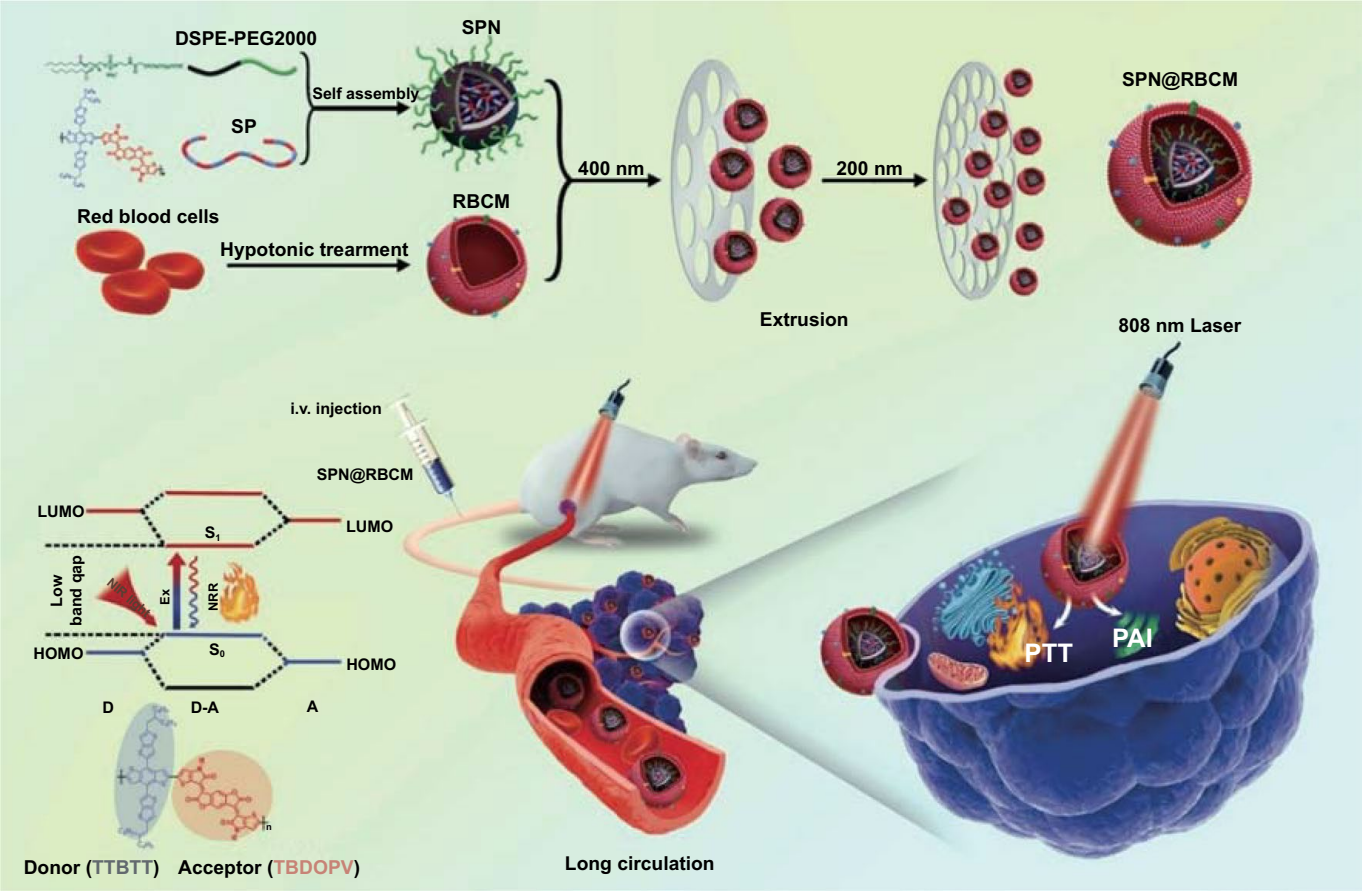
Schematic illustration for the preparation of SPN@RBCM nanoparticles and their applications in photoacoustic imaging (PAI) and photothermal therapy (PTT)

Herein, we synthesized a novel narrow bandgap donor–acceptor (D–A) conjugated polymer with thiophene-fused benzodifurandione-based oligo(p-phenylene vinylene) as the acceptor segment and thieno[3,2-b]thiophen-2-yl)benzo[1,2-b:4,5-b′]dithiophene as the donor segment, to serve as building blocks to construct SPNs for cancer phototheranostics (Scheme 1). These SPNs were further camouflaged with RBCM, namely SPN@RBCM, to reduce the rapid clearance by phagocytic cells and prolong the circulation lifetime. The D–A backbone structure endowed SPN@RBCM with excellent NIR absorption and photothermal conversion ability, which showed excellent PAI and PTT efficacy both in vitro and in vivo. In addition, owing to its ultrasmall size, SPN@RBCM had significantly deep tumor penetration and could be cleared from the body without toxicity. Compared with previously reported semiconducting polymer nanoparticles, the most distinctive feature of our SPN@RBCM is the combination of a small-sized structure and RBCM camouflage, which reconciles the dilemma between prolonged blood circulation for tumor accumulation and rapid clearance from the body to reduce long-term toxicity. Thus, this work presents a promising phototheranostic agent for clinical translation.

## Experimental Section

### Materials

Pd_2_(dba)_3_ and P(tolyl)_3_ were purchased from Sigma-Aldrich (USA). TTBDT (4,8-Bis(5-(2-ethylhexy)thieno[3,2-b]thiophen-2-yl)benzo[1,2-b:4,5-b]dithiophene-2.6-diyl)bis(trimethylstannane)) was purchased from Suna Tech Inc. (Suzhou, China). Tetrahydrofuran, methanol, dichloromethane and toluene were purchased from Sinopharm Chemical Reagent Co. Ltd. (China). 1,2-Dipalmitoyl-sn-glycero-3-phosphoethanolamine-N-[methoxy[poly(ethylene glycol)]-2000] (DSPE-mPEG2000) was purchased from A.V.T Pharmaceutical (Shanghai, China). DiO and DiR were purchased from Us Everbright Inc. (Suzhou, China). APC-affinity anti-CD11b-antibody was purchased from Thermo Fisher Scientific (USA). Hoechst 33342 was purchased from Sigma-Aldrich (USA). Cell Counting Kit-8 (CCK-8) and the Annexin V-FITC/PI apoptosis detection kit were purchased from Dojindo Laboratories (Kumamoto, Japan). Calcein AM and PI were purchased from J&K Scientific Ltd (Beijing, China). Ultrapure water was prepared by a Milli-Q Gradient System (18.2 M Ω resistivity, Millipore Corporation, Bedford, MA, USA).

### Cell Culture

SMMC-7721 cells (human hepatocellular carcinoma), QSG-7701 cells (human normal liver cells), 4T1 cells (murine breast cancer cells) and NIH-3T3 cells (murine embryonic fibroblasts cells) were all obtained from ATCC (Manassas, VA). These cells were cultured in DMEM containing 10% fetal bovine serum (FBS) in a 37 °C humidified incubator supplied with 5% CO_2_.

### Preparation of SPN@RBCM

#### Preparation of SPN

The synthetic processes and characterization of the SPs were exhibited in the supporting information (Fig. S1). Afterward, 1.0 mg of SP and 5.0 mg of DSPE-mPEG2000 were dissolved in 2 mL of THF, followed by quick addition into 20 mL of water under sonication. Afterward, the THF was removed by stirring for over 10 h at room temperature. The mixed solution was ultrafiltered at 4500 rpm for 30 min and then washed three times with pure water to remove excess DSPE-mPEG2000. Finally, the products were collected and stored at 4 °C.

#### Preparation of RBC Membrane

Whole blood was harvested from healthy BALB/c mice. Then, the red blood cells (RBCs) were separated by centrifugation at 1000 g for 10 min at 4 °C and washed 3 times with ice-cold PBS (pH = 7.4) to obtain the red blood cells. Red blood cells were ruptured by suspension in 0.25 mM PBS solution for 3 h. Afterward, the solution was centrifuged at 16,000 *g* for 30 min at 4 °C. The precipitate was repeatedly washed with ice-cold PBS and centrifuged until the RBCM turned colorless. Finally, the precipitate (RBCM) was quantified and stored at − 80 °C for future use.

#### Preparation of SPN@RBCM

SPN@RBCM was synthesized by an extrusion method. First, RBC membrane (0.2 mL, 1 mg mL^−1^) and SPN (1 mL, 1 mg mL^−1^) were mixed. Then, the mixture was extruded using an Avanti mini-syringe (Avanti, USA) through 400 nm and 200 nm polycarbonate porous membranes 15 times to obtain SPN@RBCM.

### Characterization of SPN@RBCM

SPN and SPN@RBCM were characterized by dynamic light scattering (DLS, Malvern, UK), UV–Vis-NIR spectrometry (Cary5000, Agilent), transmission electron microscopy (TEM, JEOL, Japan).

For the characterization of membrane proteins, SDS-PAGE (polyacrylamide gel electrophoresis) was used. The extracted membrane proteins of RBC vesicles and SPN@RBCM nanoparticles were detected by SDS-PAGE in a BIO-RAD electrophoresis system. Next, the resulting polyacrylamide gel was stained by Coomassie blue solution for 3–4 h to visualize the proteins. Finally, the polyacrylamide gel was washed 3–4 times with pure water before being recorded by an imaging system (BIO-RAD, USA).

### In Vitro PA Imaging

In order to test the PA imaging ability of SPN@RBCM, 1 mL of SPN@RBCM aqueous solution at different concentrations (10, 20, 30, 40, 50, and 60 μg mL^−1^) was tested with a PA instrument (Vevo3100 LAZR system, Canada).

### Photothermal Performance of SPN@RBCM

To study the temperature elevation of SPN@RBCM, 1 mL of SPN@RBCM aqueous solutions at different concentrations (10, 20, 40, and 60 μg mL^−1^) was exposed to laser irradiation (808 nm, 0.8 W cm^−2^) for 1000 s. At the same time, pure water (termed 0 μg mL^−1^ of SPN@RBCM) was exposed to the same conditions as the control sample. To further investigate the photothermal stability, 1 mL of SPN@RBCM (20 μg mL^−1^) in water was exposed to laser irradiation (808 nm, 0.8 W cm^−2^) for 1000 s and then the laser was turned off for 1000 s. This procedure was repeated four rounds. Additionally, 1 mL of ICG solution with the same treatment was set as the control group. The temperature of the solution was monitored by IR thermal camera (Ti25 Fluke Co, USA), as well as a thermocouple microprobe (*ɸ* = 0.5 mm) (STPC-510P, China) that was submerged in the solution. In addition, we compared the absorbance of SPN@RBCM and ICG before and after laser irradiation. Specifically, 1 mL of each SPN@RBCM and ICG aqueous solutions was exposed to laser irradiation (808 nm, 0.8 W cm^−2^) for different lengths of time (2.5, 5, 10, and 15 min), and the absorbance of the SPN@RBCM and ICG aqueous solutions was measured with a UV–Vis-NIR spectrometer (Cary5000, Agilent).

### Photothermal Conversion Efficiency of SPN@RBCM

The photothermal conversion efficiency (*η*) was calculated by Eqs. 1–3:

1 $$\eta = \frac{{\left( {hA\Delta T_{\hbox{max} } - Q_{s} } \right)}}{{I\left( {1 - 10^{{ - A_{\lambda } }} } \right)}}$$

2 $$\tau_{s} = \frac{{m_{D} C_{D} }}{hA}$$

3 $$\theta = \frac{\Delta T}{{\Delta T_{\hbox{max} } }}$$

where *h* is the heat transfer coefficient, *A* is the surface area of the container, *T*_max_ is the equilibrium temperature, *T*_Surr_ is the surrounding temperature, Δ*T*_max_ = *T*_max_ − *T*_Surr_, *I* is the incident laser power (0.8 W cm^−2^), and *A*_λ_ is the absorbance of 20 μg mL^−1^ SPN@RBCM at 808 nm. *Q*_s_ is the heat associated with the light absorbance of the solvent, which is measured independently to be 25.2 mW by using pure water. The value of *hA* is derived from Eq. 2. *τ*_*s*_ is the time constant of the sample system, *m*_*D*_ and *C*_*D*_ are the mass and heat capacity of pure water (*m*_D_ = 1 g, *C*_D_ = 4.2 J g^−1^). Combining Eq. 2 with Eq. 3, *τ*_*s*_ could be determined by applying the linear time data from the cooling period versus − ln*θ*.

$$\begin{aligned} & \tau_{S} = 284.87;\quad hA = \frac{{m_{D} C_{D} }}{{\tau_{S} }} = \frac{4.2J}{284.87} = 0.0147; \\ & A_{\lambda } = 0.545;\quad I = 0.8\,{\text{W}}\,{\text{cm}}^{ - 2} ;\quad \Delta T_{\hbox{max} } = 24.6\,^{\circ } {\text{C}} \\ \end{aligned}$$

$$\eta = ((4.2/284.8686) \times 24.6 - 0.0252) \times 100\% /\left( {0.8 \times \left( {1 - 10^{ - 0.545} } \right)} \right) = 0.3375/0.5719 \, \times 100\% = 59.01\%$$

### In Vitro Cytotoxicity Assay of SPN and SPN@RBCM

The cytotoxicity of SPN and SPN@RBCM was evaluated in cancer cell lines (SMMC-7721 cells and 4T1 cells) and normal cell lines (QSG-7701 cells and NIH-3T3 cells) by CCK-8 assay. The cells were seeded in a 96-well plate at a density of 1.5 × 10^4^ cells per well and incubated for 12 h. The original medium was removed, and then fresh medium containing different concentrations of SPN or SPN@RBCM (10, 20, 30, 40, 50, or 60 μg mL^−1^) was added. Meanwhile, untreated cells were used as a control. After 24 h of incubation, the medium containing different concentrations of SPN or SPN@RBCM was removed, and the cells were washed twice with PBS. Then, 100 μL fresh medium with 10% CCK-8 solution was added. After 1 h of incubation at 37 °C, the absorbance at 450 nm of each well was measured with a microplate reader (Molecular Devices, USA). Cell viability was calculated as follows:

$${\text{Cell}}\,{\text{viability}}\,\left( \% \right)\, = \,\left( {A_{1} - A_{\text{blank}} } \right)/\left( {A_{0} - A_{\text{blank}} } \right)\, \times \,100\%$$

where *A*_1_ is the absorbance value of the cells treated with different concentrations of SPN@RBCM, *A*_0_ is the absorbance value of cells without treatment, and the *A*_blank_ is the absorbance value of the CCK-8 medium solution itself at 450 nm. All samples were performed in four parallel repeats.

### In Vitro Photothermal Therapy

To investigate the PTT efficiency of SPN@RBCM, SMMC-7721 cells or 4T1 cells were seeded in a 96-well plate at a density of 1.5 × 10^4^ and cultivated for 12 h. The original medium was replaced by fresh medium containing different concentrations of SPN@RBCM (10, 20, or 30 μg mL^−1^) and further incubated for another 8 h. Afterward, the cells were irradiated with an 808 nm laser (0.8 W cm^−2^, 10 min) and cultivated for 24 h at 37 °C with 5% CO_2_. Finally, the cells were washed 3 times with PBS and incubated with fresh medium containing 10% CCK-8 solution to measure the cell viability according to the above method.

Live and dead cell assay and apoptosis assay were also utilized to prove the PTT efficiency. SMMC-7721 cells were seeded at a density of 1.5 × 10^4^ in a 96-well plate and cultivated for 12 h. Next, 60 μg mL^−1^ SPN@RBCM was added with fresh medium and incubated for another 8 h. Afterward, the cells were irradiated with an 808 nm laser (0.8 W cm^−2^, 10 min). Subsequently, the treated cells were stained with calcein AM and PI and imaged under a fluorescence microscope (Zeiss Axio Vert. A1, Germany). For the apoptosis assay, the cells were treated as described above and then stained with Annexin V-FITC/PI solution. Finally, the treated cells were detected by flow cytometry (BD FACSAria TM III, USA).

### In Vivo PA Imaging of SPN@RBCM

The BALB/c mice were obtained from China Wushi, Inc. (Shanghai, China). The animal experiments were strictly carried out following the “National Animal Management Regulations of China” and approved by the Animal Ethics Committee of the Mengchao Hepatobiliary Hospital of Fujian Medical University. Tumor-bearing mice were established by subcutaneously injection of 4T1 cells at a density of 1 × 10^6^ per mouse. Afterward, when the tumor size reached 50 mm^3^, SPN or SPN@RBCM (100 µL, 5 mg mL^−1^) was injected into the 4T1 tumor-bearing mice through the tail vein, and the in vivo PA imaging was monitored by a PA instrument (Vevo3100 LAZR system, Canada) with a laser wavelength of 808 nm 24 and 48 h postinjection.

### Biodistribution of SPN@RBCM

For the in vivo biodistribution investigation, 4T1 tumor-bearing mice were i.v. injected with DiR-labeled SPN or SPN@RBCM (100 μL, 5 mg mL^−1^), and then an IVIS imaging system was used to monitor the fluorescent signals at the tumor site 0, 2, 4, 6, 8, 10, 24, and 48 h postinjection. Afterward, the mice were killed, and the main organs were resected and imaged by the IVIS imaging system. In order to study the penetration capacity of SPN@RBCM in vivo, the tumor tissue was further cut into three pieces and imaged by the IVIS imaging system.

### Blood Circulation

The DiR-labeled SPN@RBCM nanoparticles were i.v. injected into healthy SD rats at a dose of 5 mg kg^−1^. SPN without RBCM coating were used as the control. At different time points of injection (2, 6, 12, 24, and 48 h), 20 μL of blood was collected from the mouse tail and centrifuged immediately at 3000 rpm for 10 min to obtain serum, and the mice were imaged by an IVIS imaging system to detect the fluorescence intensity.

### In Vivo Uptake by Macrophages

The DiO-labeled SPN@RBCM or SPN nanoparticles were i.v. injected into BALB/c mice with an equal SPN-based dose of 5 mg kg^−1^. Then, 500 of μL blood was withdrawn from the mice 4 h postinjection. Afterward, the blood samples were diluted twofold with ice-cold PBS, and Ficoll-Paque premium sterile solution was utilized to obtain monocytes. Subsequently, the monocytes were cultivated with APC-affinity anti-CD11b antibody (3:500) for 30 min at room temperature. Then, the stained cells were washed with PBS and centrifuged, of which this procedure was repeated 3 times (800 g, 5 min), followed by suspension in PBS. Finally, the sample was analyzed by flow cytometry analysis (BD FACSAria TM III, USA).

### In Vivo Infrared Thermal Imaging

4T1 tumor-bearing mice received SPN@RBCM administration through tail vein injection at a dose of 5 mg kg^−1^. After 24 h, the tumor sites of the mice were irradiated by an 808 nm laser (0.5 W cm^−2^) for 10 min, and the photothermal performance of SPN@RBCM in the tumor site was observed by an IR thermal camera (Ti25 Fluke Co, USA).

### In Vivo Antitumor Effect

To study the photothermal therapy efficiency, 4T1 tumor-bearing mice were randomly divided into 5 groups (*n* = 5, each group), and SPN or SPN@RBCM (100 μL, 5 mg mL^−1^) was injected into each mouse through the tail vein. In detail, the groups were divided as follows: (1) treatment with PBS; (2) treatment with PBS and laser irradiation (808 nm, 0.5 W cm^−2^) for 10 min; (3) treatment with SPN@RBCM nanoparticles; (4) treatment with SPN and laser irradiation (808 nm, 0.5 W cm^−2^) for 10 min; and (5) treatment with SPN@RBCM and laser irradiation (808 nm, 0.5 W cm^2^) for 10 min. Laser irradiation was implemented 24 h after injection. PTT efficacy was evaluated by measuring the body weights and tumor volumes of each group every 2 days. The tumor volume was calculated by the formula: Tumor volume = length × width^2^/2. The length and width were measured by Vernier caliper.

To examine the histological changes in the tumors, one tumor-bearing mouse in each group was killed 2 days after treatment. The tumor was collected and fixed with formalin solution, embedded in paraffin and sliced for histological examination (H&E, Ki67 and TUNEL staining).

In order to assess the long-term biosafety of SPN@RBCM, the treated mice were killed and the major organs were harvested (heart, liver, spleen, lung, and kidney), and then fixed with formalin solution for H&E examination. We also evaluated the toxicity of SPN@RBCM by routine blood and serum biochemistry analyses. After treatment for different lengths of time (0, 1, and 7 days), the blood of the SD rats was collected in EDTA2K spray-coated tubes and analyzed using an automated hematology analyzer.

### Statistical Analysis

The results of the experiments are reported as the mean ± standard deviation. The different experimental data of each group were analyzed by ANOVA with post hoc tests using Prism 6 software (GraphPad). Different significance levels of the data were considered at *p < 0.05, **p < 0.01, ***p < 0.001.

## Results and Discussion

### Preparation and Characterization of SPN and SPN@RBCM

The D–A-conjugated semiconducting polymer (SP) was synthesized though Stille coupling polymerization from thiophene-fused benzodifurandione-based oligo(p-phenylene vinylene) (TBDOPV, as electron acceptor) and thieno[3,2-b]thiophen-2-yl)benzo[1,2-b:4,5-b′]dithiophene (TTBDT, as electron donor), similar to previous literature [20]. The detailed synthetic route and characterization are displayed in Figs. S1–S6. Hydrophobic SP was then assembled with DSPE-mPEG2000 to construct hydrophilic nanoparticle (SPN) through nanoprecipitation method. To coat the RBCM onto the surface of SPN, freshly prepared RBCM-derived vesicles were coextruded with SPNs through 400 and 200 nm polycarbonate porous membranes (Scheme 1), respectively.

**Fig. 1:**
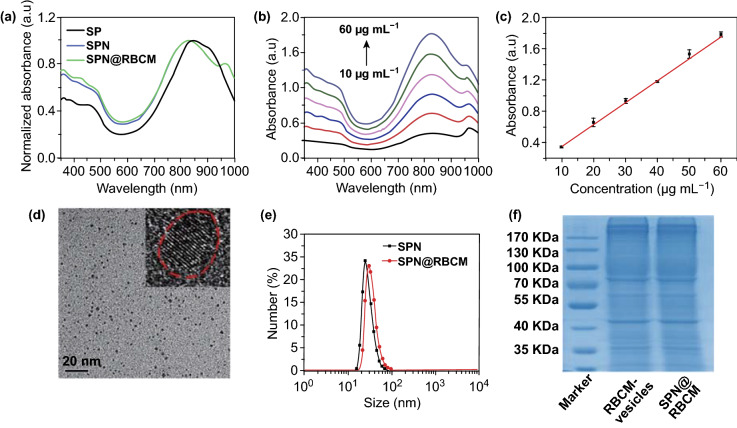
**a** Vis–NIR spectra of SP in THF, SPN and SPN@RBCM in PBS. **b** Vis–NIR spectra of SPN@RBCM in PBS at various concentrations ranged from 10 to 60 μg mL^−1^. **c** Corresponding absorbance of SPN@RBCM at 808 nm in **b**. **d** TEM image of SPN, the inset picture is the enlarged image of a single nanoparticle, while the red dashed circle indicated its crystal structure. **e** DLS results of SPN and SPN@RBCM. **f** SDS-PAGE electrophoresis patterns of RBCM vesicles and SPN@RBCM

Next, the physicochemical properties of SP, SPN, and SPN@RBCM were systematically investigated. As shown in Fig. 1a, the UV–Vis-NIR absorbance of SP in THF displays a strong absorption peak at 840 nm corresponding to the intramolecular charge transfer from the D to A. SPN in PBS showed a similar absorption pattern to that of SP in THF, confirming that the strong NIR absorption does not come from J-aggregation [47]. The calculated molecular orbitals of the SP dimer showed that both the HOMO and LUMO were delocalized along the polymer backbone, indicating a large π-conjugated system with a narrow bandgap of 1.75 eV (Fig. S7), which was consistent with the NIR absorption measurements. In addition, the characteristic absorption of SPN@RBCM remained nearly unchanged compared to SPN before RBCM coating. To evaluate the NIR absorption capacity, the corresponding mass extinction coefficient at 808 nm was further investigated, which was measured to be 28.3 L mg^−1^ cm^−1^ (Fig. 1b, c). The morphologies of SPN and SPN@RBCM were observed by transmission electron microscopy (TEM). As shown in Fig. 1d, SPN exhibited a uniform spherical shape with a small size of approximately 2–5 nm, and the crystal pattern from the high-resolution TEM image further verified its P-dot structure. In contrast, SPN@RBCM presented an obvious core–shell structure, which is in good agreement with the reported membrane width of erythrocytes (Fig. S8) [25, 26]. Dynamic light scattering (DLS) measurements revealed an average hydrodynamic size (*D*_h_) of 74.6 ± 1.2 nm with a polydispersity index (PDI) value of 0.386, indicating the monodisperse tendency of SPN@RBCM in aqueous solution, which was slightly larger than SPN before RBCM coating (*D*_h_: 50.2 ± 0.3 nm; PDI: 0.345) (Fig. 1e). The larger size determined by DLS than by TEM was most likely due to the nanoparticles under different dry (for TEM) and wet (for DLS) measurement conditions, as well as slight aggregation in solution [48]. Additionally, SPN@RBCM did not show obvious aggregation over 14 days in PBS with 10% FBS, indicating its excellent colloid stability under physiological conditions (Fig. S9). In addition, zeta potential analysis showed that the charge of SPN changed from − 42.3 ± 0.5 to − 34.8 ± 0.8 mV after RBCM coating, which is similar to the level of RBC vesicles (− 33.1 ± 0.3 mV) (Fig. S10). The SDS-PAGE analysis demonstrated that no major difference in the protein profile was observed between SPN@RBCM and the sourced RBCM vesicles (Fig. 1f). The loading capacity of SPN and RBCM in SPN@RBCM was determined to be 67.3% and 32.7%, respectively, according to NIR absorption measurement. These results confirmed that the RBCM was successfully coated on SPN.

### Photothermal Performance of SPN@RBCM

**Fig. 2:**
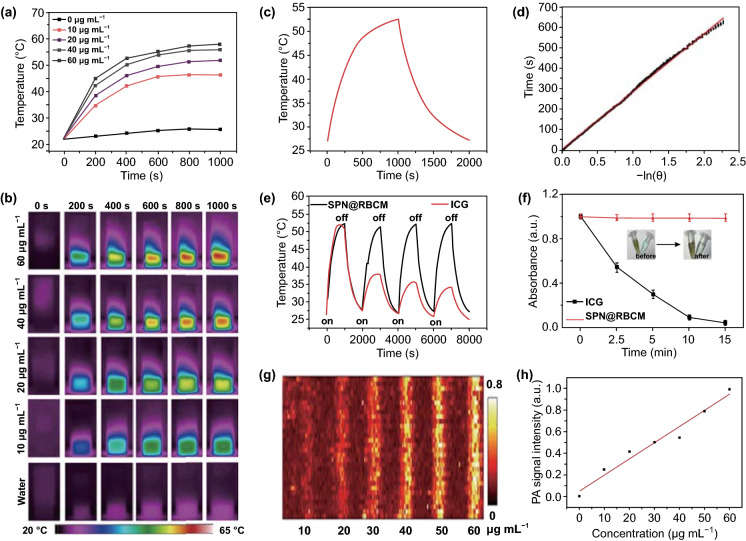
**a**, **b** Temperature elevation of SCS@RBCM as a function of concentration for 1000 s, with a laser power density of 0.8 W cm^−2^. **c** Photothermal performance of SCS@RBCM with laser irradiation for 1000 s (0.8 W cm^−2^) and then the laser was shut off. **d** Linear time data *versus* -ln θ obtained from the cooling period of Fig. 2d. **e** Photothermal stability of SCS@RBCM and ICG, upon four ‘‘On-to-Off’’ laser cycles. **f** Normalized absorbance of SCS@RBCM and ICG after irradiation with different times (0.8 W cm^−2^). **g** In vitro PA images of SCS@RBCM with different concentrations ranged from 10 to 60 µg mL^−1^. **h** Linear dependence between the PA signals and concentrations of SPN@RBCM

Considering the strong NIR absorption of SPN@RBCM, we further explored their photothermal conversion ability, which is a vital perquisite for tumor PAI and PTT. As shown in Fig. 2a, the temperature of SPN@RBCM aqueous solution increased with the extension of irradiation time, and the concentration increased upon 808 nm laser irradiation. For instance, as the concentration increased from 10 to 60 μg mL^−1^, the temperature of the SPN@RBCM solution increased from 22 to 58 °C after 1000 s of laser irradiation (0.8 W cm^−2^), while the temperature of pure water (0 μg mL^−1^ of SPN@RBCM) changed insignificantly. The photothermal conversion of SPN@RBCM solution was also observed by the infrared (IR) thermal images (Fig. 2b). The photothermal conversion efficiency of SPN@RBCM was calculated to be 59.01%, according to a previously reported method (Fig. 2c, d) [49], which is significantly higher than that of Au NRs (21%) [50], CuS nanocrystals (25.7%) [51], black phosphorus (28.4%) [52], and melanin nanospheres (40%) [53]. Furthermore, compared with other reported semiconducting polymer nanoparticles, such as SPN1-C (37%) [54] and DPP-IID-FA NPs (49.5%) [49], our nanoparticles displayed significantly higher photothermal conversion efficiency.

In addition to photothermal conversion efficiency, photothermal stability is another important index in photothermal therapy. To investigate this parameter, SPN@RBCM was irradiated by the 808 nm laser for four “on-and-off” cycles. Each cycle consisted of 1000 s of irradiation (on state) and then turned off to cool to room temperature (off state). ICG, an FDA-approved NIR dye with photothermal performance under 808 nm laser irradiation was selected as a control. Compared with ICG, which lost nearly all of its photothermal conversion ability, there was no appreciable decline in temperature elevation for SPN@RBCM (Fig. 2e). This phenomenon could be explained by the serious photobleaching of ICG molecules under strong 808 nm laser irradiation, while the NIR absorption ability of SPN@RBCM did not change under the same condition (Fig. 2f). The PAI performance of SPN@RBCM at 808 nm was determined at different concentrations ranging from 0 to 60 μg mL^−1^, showing that its PA signals increased linearly with increasing concentration (Fig. 2g, h). Overall, these results suggest the high and stable photothermal performance of SPN@RBCM, which can authorize its great potential in PAI and PTT treatment.

### In Vitro Cytotoxicity Assay

Cytotoxicity was evaluated in a series of cell types including normal cells (QSG-7701, NIH-3T3) and cancer cells (SMMC-7721, 4T1), by using the CCK-8 assay. Cell viability values (%) were assessed by the CCK-8 proliferation test versus incubation with different concentrations of SPN or SPN@RBCM in living cells for 24 h. As shown in Fig. S11, after coating with RBCM, the cytotoxicity of SPN was significantly reduced. Specifically, in NIH-3T3 and 4T1 cells, the viability was above 90% even at a high concentration of SPN@RBCM (60 μg mL^−1^), but dramatically decreased to below 40% for the same concentration of SPN. These results indicate that the RBCM decoration can improve the biocompatibility of SPN for biological applications.

### In Vitro Photothermal Therapy

**Fig. 3:**
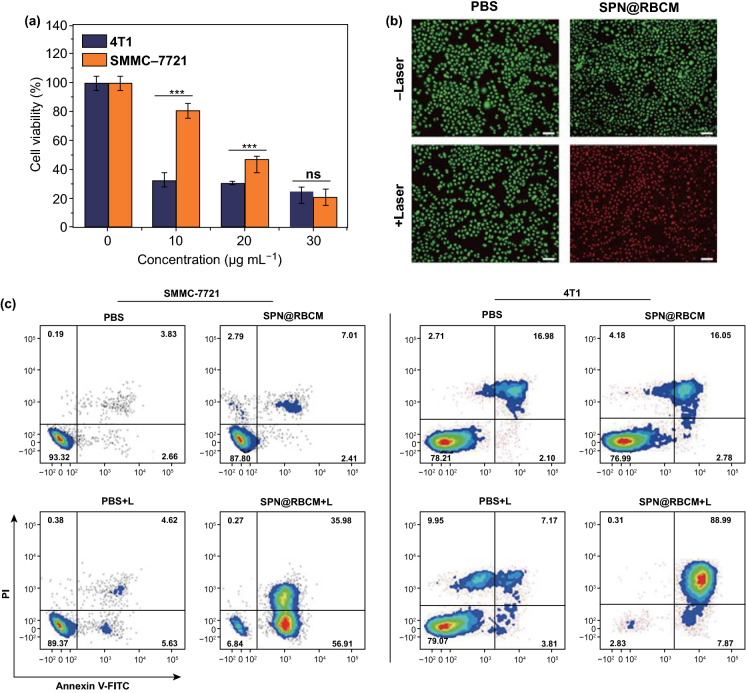
The in vitro therapeutic efficacy evaluated by CCK-8 assay (*p < 0.05, **p < 0.01, and ***p < 0.001; *n* = 4 per group) (**a**), live/dead assay (**b**), Scale bar: 200 μm, and Annexin V-FITC/PI apoptosis assay (**c**)

The excellent photothermal performance of SPN@RBCM motivates us to further explore its feasibility in the ablation of cancer cells. To this end, SMMC-7721 or 4T1 cells were incubated with SPN@RBCM at different concentrations and then irradiated by an 808 nm laser at 0.8 W cm^−2^ for 10 min, and the cell viability was quantified by CCK-8 assay. As shown in Fig. 3a, viability of SMMC-7721 cells decreased as the SPN@RBCM concentration increased from 0 to 30 μg mL^−1^. Moreover, compared with SMMC-7721 cells, 4T1 cells were more susceptible to PTT with only 30% surviving even at a very low dose of 10 μg mL^−1^. The cell status after PTT treatment was also observed by calcein AM (living cells with green fluorescence) and propidium iodide (PI, dead cells with red fluorescence) staining (Fig. 3b). The cells treated only with SPN@RBCM (60 μg mL^−1^) or only with laser irradiation showed entirely green fluorescence, indicating no obvious dark cytotoxicity of SPN@RBCM or phototoxicity from laser irradiation alone. However, with the incorporation of SPN@RBCM treatment and laser irradiation, the cell viabilities sharply decreased for both SMMC-7721 and 4T1 cells, which was in good agreement with the CCK-8 results. Moreover, the PTT anticancer effect was also determined by flow cytometry using the Annexin V-FITC/PI apoptosis assay (Fig. 3c). Compared with other groups, the highest percent of apoptosis (93% in SMMC-7721 cells and 97% in 4T1 cells) was found in the cells treated with SPN@RBCM and laser irradiation. Collectively, these results suggest that SPN@RBCM have great potential as a highly efficient PTT agent with excellent biocompatibility for cancer treatment.

### In Vivo Distribution and PAI of SPN@RBCM

**Fig. 4:**
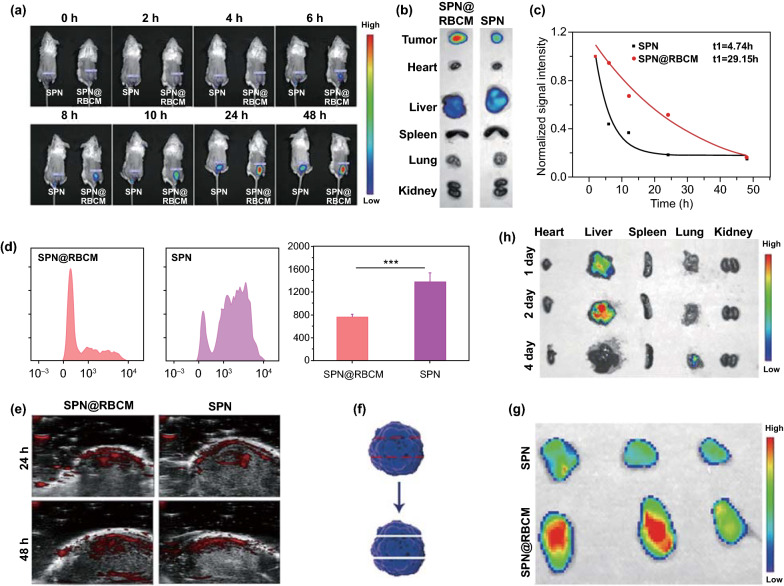
**a** In vivo fluorescence imaging of 4T1 tumor-bearing mice as a function of time after intravenous injection of DiR-loaded SPN or SPN@RBCM. **b** Ex vivo fluorescence imaging of tumor and major organs harvested after 48 h of intravenous injection. **c** Normalized fluorescence intensity of SPN or SPN@RBCM in the serum at different time points after intravenous injection. **d** In vivo uptake of DiO-loaded SPN and SPN@RBCM by Macrophages which was analyzed by FACS. **e** In vivo PA imaging of 4T1 tumor-bearing mice as a function of time after intravenous injection of SPN or SPN@RBCM. **f** Schematic illustration of tumor tissue cut into three slices for fluorescence imaging. **g** Ex vivo fluorescence imaging of tumor slices. **h** Time-dependent ex vivo fluorescence imaging of major organs

To investigate the tumor accumulation and in vivo distribution of nanoparticles, BALB/c mice were subcutaneously injected with 4T1 cancer cells to establish a tumor-bearing mouse model. Afterward, a near-infrared fluorescence probe (DiR) was loaded into SPN and SPN@RBCM with the nanoprecipitation procedure mentioned in Sect. 2.3 for in vivo fluorescence imaging (FL) after intravenous injection. As shown in Fig. 4a, the DiR fluorescence signal clearly emerged in the tumor site after injection with the nanoparticles, and these signals gradually increased over time, ranging from 0 to 24 h, and then reached a platform that was sustained for more than 48 h, suggesting effective accumulation within tumor via passive targeting mechanisms such as the EPR effect. Moreover, we examined DiR fluorescence in the excised major organs of treated mice 48 h postinjection (Fig. 4b). In addition to the notable fluorescence that appeared in the liver, which indicates the inevitable capture of SPN@RBCM by the reticuloendothelial system (RES), the accumulation of SPN@RBCM in the tumor site was significantly high. Meanwhile, compared with SPN, SPN@RBCM showed enhanced tumor accumulation while reduced liver capture. Taken together, these observations indicate that the RBC membrane favors tumor accumulation and escapes from the RES capture.

It is well known that the longer in vivo circulation lifetime of NPs could contribute to the EPR effect for improving tumor accumulation [55]. Thus, we checked whether SPN@RBCM exhibited superior blood retention compared to SPN after i.v. injection. At different postinjection times, 20 μL of blood was collected from the tail vein to determine NP contents by fluorescence measurements. The results showed that SPN@RBCM exhibited observably enhanced blood retention compared with SPN (Fig. 4c), implying that SPN@RBCM can inherit the merit of long circulation lifetime from sourced RBCs. We further explored the potential mechanism by which elimination was reduced to improve the blood retention of SPN after RBCM coating. As macrophage internalization is the major pathway for NP elimination in the RES system, we next checked the in vivo NP clearance by macrophages after i.v. injection. For this purpose, monocytes were isolated from the blood, and then incubated with APC-affinity anti-CD11b antibody. The engulfment of NPs by macrophages could be analyzed according to the DiO signal in CD11b positive cells. As shown in Fig. 4d, SPN@RBCM showed improved anti-phagocytosis ability compared to uncoated SPN in vivo, indicating decreased recognition by immune system after RBCM camouflage.

Efficient tumor accumulation was also validated through the in vivo PAI signal of tumor in living mouse after i.v. injection of SPN@RBCM. As shown in Fig. 4e, the tumor showed obvious PA signals at 808 nm after 24 and 48 h of injection, and the PA intensity of SPN@RBCM-injected mice was stronger than that of SPN injected mice, which was in line with the fluorescence imaging results.

In addition to high tumor accumulation, the small size of SPN@RBCM might also contribute to enhanced tumor penetration. To verify this hypothesis, the tumor excised from the mice administrated with SPN@RBCM was cut into three slices from top to bottom, as illustrated in Fig. 4f, and fluorescence images of each slice were recorded. As shown in Fig. 4g, the DiR signal appeared in the whole region of every slice from SPN@RBCM-treated mice, suggesting the good tumor penetration of SPN@RBCM in vivo. Furthermore, small-sized nanoparticles can avoid the potential long-term retention and toxicity. Accordingly, we investigated the clearance route and biodistribution of SPN@RBCM in normal BALB/c mice. Time-dependent ex vivo fluorescence imaging of the major organs revealed that SPN@RBCM was mainly located in the liver with the first 2 days after intravenous injection. However, after 4 days, no FL signal was detected in any of the organs, indicating favorable clearance of SPN@RBCM in vivo (Fig. 4h).

### In Vivo Photothermal Therapy

**Fig. 5:**
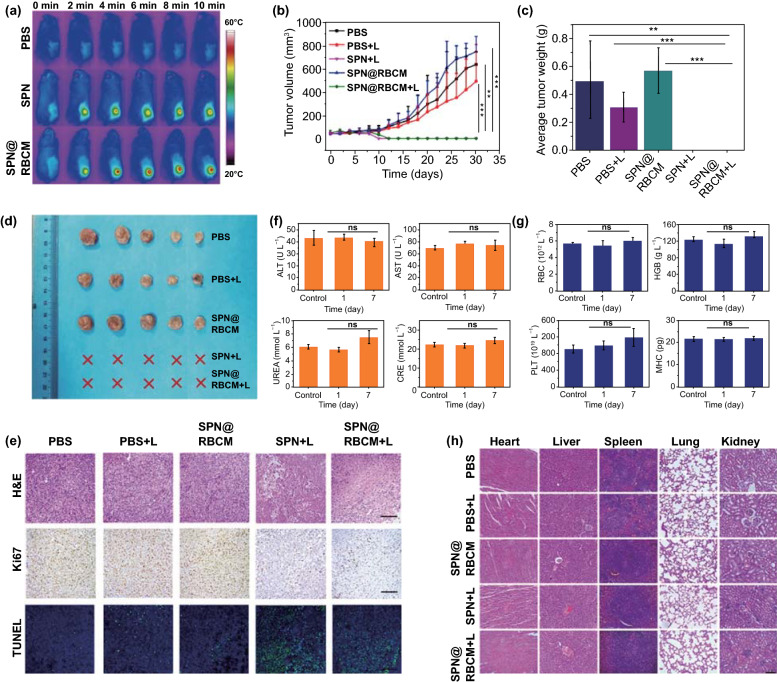
**a** IR thermal images of 4T1 tumor-bearing BALB/c mice under 808 nm laser irradiation (808 nm, 0.5 W cm^−2^) after PBS, SPN or SPN@RBCM injection. **b**–**d** Tumor growth curves (**b**), average tumor weight (**c**) and tumor photograph (**d**) after i.v. injection of PBS, SPN, and SPN@RBCM with or without 808 nm laser irradiation (*p < 0.05, **p < 0.01, and ***p < 0.001; *n* = 5 per group). **e** Optical microscopy images of tumor slices stained with H&E, antigen Ki67 and TUNEL after various treatments as indicated above. Scale bar: 100 μm. **f**, **g** Blood biochemistry (**f**) and routine indexes (**g**) of the BALB/c mice after i.v. injection of SPN@RBCM. **h** Histological H&E staining of major organs after various treatments as indicated above. Scale bar: 100 μm

The excellent in vitro cell-killing effect of SPN@RBCM nanoparticles under NIR irradiation further encouraged us to explore their in vivo PTT antitumor efficacy. For this purpose, 4T1 tumor-bearing mice were randomly divided into five groups (PBS, PBS + Laser, SPN@RBCM, SPN + Laser, SPN@RBCM + Laser). As shown in Fig. 5a, the temperature of the tumor site in the mice that received i.v. injection of SPN rapidly reached 50 °C with laser irradiation (808 nm, 0.6 W cm^−2^) for 10 min, verifying the efficient photothermal conversion ability of SPN in the tumor site under laser irradiation. More significantly, under the same laser irradiation conditions, the temperature of the tumors in the mice injected with SPN@RBCM reached 56 °C within 10 min due to the higher tumor accumulation after coated with RBCM. In contrast, the temperature of tumor in PBS, PBS + Laser or SPN@RBCM groups changed slightly under laser irradiation. Afterward, the PTT therapeutic efficacy of our nanoparticles was investigated by observing the changes of tumor size after laser irradiation. As shown in Fig. 5b, the tumor sizes in the PBS, PBS + Laser, and SPN@RBCM groups experienced rapid growth, indicating that single laser irradiation or SPN@RBCM treatment alone have no effect on tumor inhibition. However, upon laser irradiation, both SPN and SPN@RBCM could restrain tumor growth completely, proving the remarkable PTT antitumor efficacy of our nanoparticles because the temperature of the tumors in these two groups increased above 42 °C, which is sufficient to kill cancer cells. Meanwhile, the tumor weights were also recorded to confirm the antitumor efficacy at the end of treatment on day 30 (Fig. 5c). The average tumor weight in PBS group, with or without laser irradiation, was above 0.3 g, and the SPN@RBCM group without laser irradiation showed a similar result. Nevertheless, SPN and SPN@RBCM with laser irradiation had no tumor residues. These results were also confirmed by the photographs of the tumor tissues at the end of treatment (Fig. 5d), further suggesting the optimal PTT antitumor efficacy of SPN@RBCM. Besides, the body weights of each group had no significant changes during the monitoring period, indicating much less biotoxicity of our prepared nanoparticles (Fig. S12). Then, histological examination (H&E, Ki67 and TUNEL) of the treated tumor also was utilized to confirm the PTT antitumor efficacy. As shown in Fig. 5e, consistent with the above antitumor evaluations, SPN@RBCM with laser irradiation caused serious damage to tumor tissues, compared with the control groups. Although there were no significant differences in the antitumor effects between SPN and SPN@RBCM in these experiments, we still considered that the RBCM coating was meaningful, considering that this strategy could increase SPN tumor accumulation, which might in return reduce the administration dose or laser power to improve the safety of PTT treatment.

Subsequently, we analyzed the blood biochemistry and routine indexes to evaluate the potential toxicity of SPN@RBCM in vivo (Fig. 5f, g). All the tested results were within the normal range after intravenous administration at different time points. The histopathological changes of the major organs were observed for all groups at the end of treatment (Fig. 5h). There were no significant physiological abnormalities in the major organs of all groups. Overall, these results suggest the low systemic toxicity of prepared nanoparticles. In addition, the safety of laser irradiation is another important index for the clinical translation of PTT. Although the laser power used in our current study (0.5 W cm^−2^) is still over the safety limit (maximum permissible exposure of 0.33 W cm^−2^), this issue could be resolved by increasing the administration dose to reach the comparable PTT efficacy at lower laser photo-density.

## Conclusions

In summary, we have prepared a novel phototheranostic platform based on D–A semiconducting polymer nanoparticle (SPN) with surface camouflaged by red blood cell membrane (RBCM) for NIR photoacoustic imaging (PAI) and photothermal therapy (PTT). The as-prepared SPN@RBCM nanoparticles display excellent NIR absorbance, high photothermal conversion ability, and good photothermal stability. Moreover, inherited from the natural features of the sourced red blood cells, the SPN@RBCM nanoparticles possess excellent performance of the enhanced biocompatibility, reduced retention in the reticuloendothelial system, prolonged blood circulation, and the improved tumor accumulation. On the other hand, the SPN@RBCM has small size (< 5 nm), which not only shows deep penetration in tumor site, but also can be easily cleared out from the body to overcome the long-term toxicity induced by the undefined residues after systemic administration. Therefore, as-prepared SPN@RBCM could act as an optimal phototheranostic nanoagent to provide intensified PA signals for tumor imaging and enhanced photothermal killing effects for cancer treatment. Given these outstanding characteristics, this nanoplatform might be a promising phototheranostic agent for clinical translation in future.

## Electronic supplementary material

Below is the link to the electronic supplementary material.

Supplementary material 1 (PDF 1034 kb)
