# Supplementary material for: RBC Membrane Camouflaged Semiconducting Polymer Nanoparticles for Near-Infrared Photoacoustic Imaging and Photothermal Therapy
Source: Nanomicro Lett. 2020 Apr 20;12:94. doi: 10.1007/s40820-020-00429-x (PMC7770914; doi:10.1007/s40820-020-00429-x)
Supplement: Supplementary file 1 — Supplementary material 1 (PDF 1034 kb) [file 40820_2020_429_MOESM1_ESM.pdf]

Supporting Information for

RBC Membrane Camouflaged Semiconducting Polymer Nanoparticles for Near-Infrared Photoacoustic Imaging and Photothermal Therapy

Dongye Zheng[1, 2, 3], Peiwen Yu[2, 4], Zuwu Wei [2, 4], Cheng Zhong[5], Ming Wu [2, 4, *], Xiaolong Liu[1, 2, 3, 4, *]

[1]School of Life Sciences, Fujian Agriculture and Forestry University, Fuzhou 350002, People's Republic of China

[2]The United Innovation of Mengchao Hepatobiliary Technology Key Laboratory of Fujian Province, Mengchao Hepatobiliary Hospital of Fujian Medical University, Fuzhou 350025, People's Republic of China

[3]Key Laboratory of Design and Assembly of Functional Nanostructures, Fujian Institute of Research on the Structure of Matter, Chinese Academy of Sciences, Fuzhou 350002, People's Republic of China

[4]Mengchao Med-X Center, Fuzhou University, Fuzhou 350116, People's Republic of China

[5]Department of Chemistry, Hubei Key Lab on Organic and Polymeric Optoelectronic Materials, Wuhan University, Wuhan 430072, People's Republic of China

Dongye Zheng and Peiwen Yu contributed equally to this work

*Corresponding authors. E-mail: wmmj0419@163.com (Ming Wu); xiaoloong.liu@gmail.com (Xiaolong Liu)

S1 Synthesis and Characterization

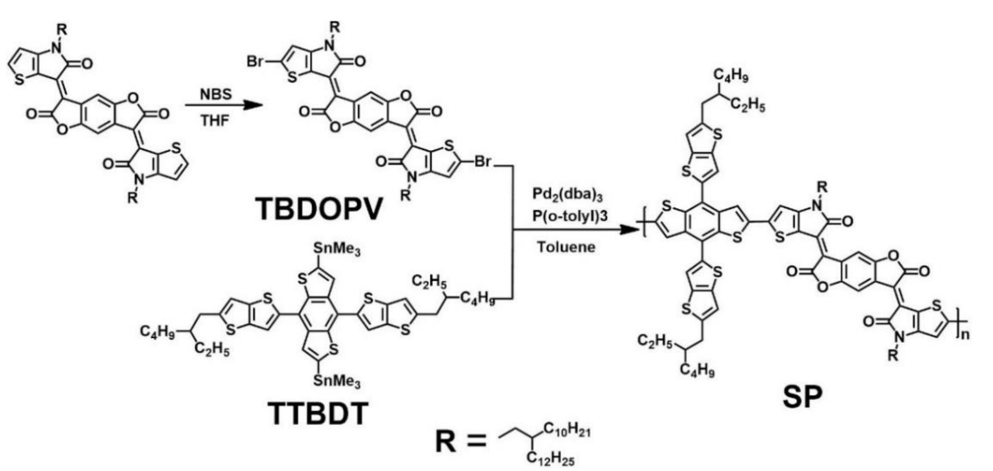

Fig. S1 Synthetic procedure for D-A conjugated semiconducting polymer (SP)

The overall synthesis route was illustrated in Fig. S1. TTBDT was commercially purchased from Suna Tech Inc. (Suzhou, http://www.sunatech.com.cn/). TBDOPV was synthesized according to the previous report [S1]. The synthesis procudure of SP were illustrated in Fig. S1.

S2 General Procedure for SP

In Schlenk tube, the TBDOPV (0.1158 g, 0.122 mmol), TTBDT (0.0673 g, 0.122 mmol), $Pd_2(dba)_3$ (0.0034g, 0.0037 mmol), and P(o-tolyl)$_3$ (0.0045 g, 0.0146 mmol) were mixed in toluene (10 mL) under nitrogen. The resulting mixture was subjected to three cycles of evacuation and admission of nitrogen. After stirred at 110 °C for 72 h, the solution was cooled down to room temperature, and then poured into stirring methanol to precipitate the polymer product. The precipitated polymer product (SP) was collected by filtration and purified with methanol and DCM in Soxhlet extractions for 24h, respectively. The final polymer product as a blackish green solid was collected.

S3 Characterization of TBDOPV

^{1}H NMR ($CDCl_3$, ppm): δ 8.86 (s, 2H), 6.76 (s, 2H), 3.63-3.62 (d, 4H), 1.81 (m, 2H), 1.33-1.24 (m, 80H), 0.87-0.85 (t, 12H). ^{13}C NMR ($CDCl_3$, ppm): δ 169.61, 169.06, 154.27, 151.20, 130.60, 129.76, 125.22, 116.22, 115.87, 115.03, 109.19, 46.31, 37.19, 31.93, 31.45, 29.96, 29.71, 29.66, 29.60, 29.38, 29.36, 26.42, 22.70, 14.13.

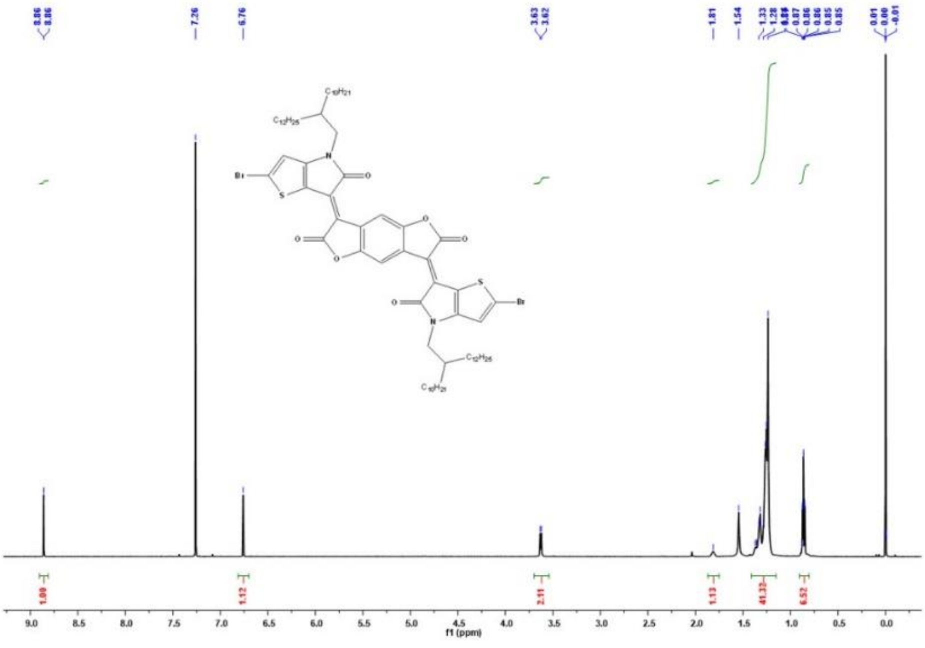

Fig. S2 ^{1}H NMR spectrum of TBDOPV

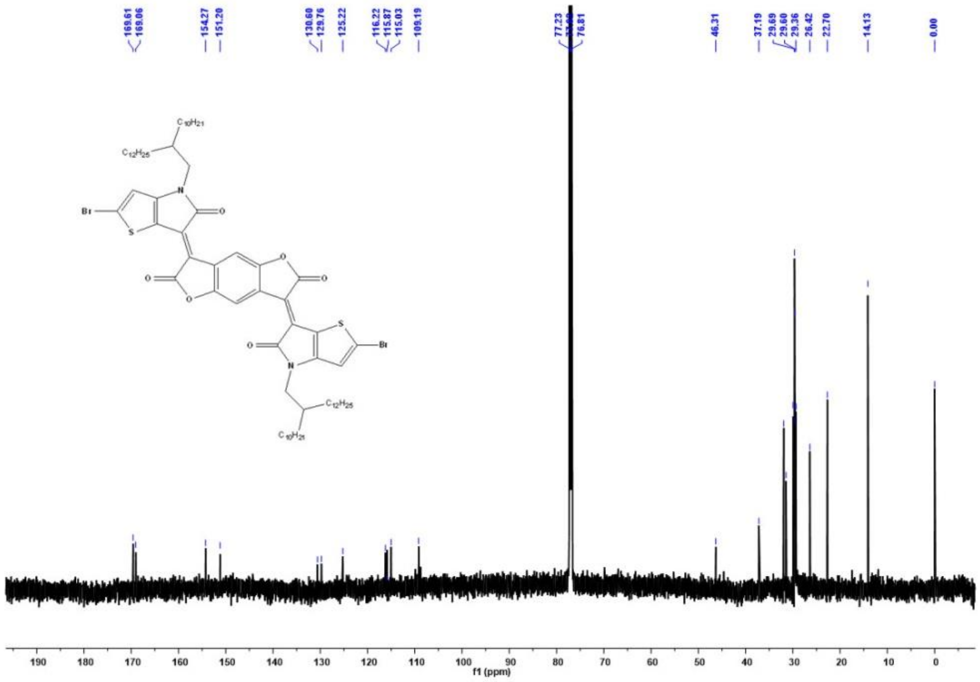

Fig. S3 ^{13}C NMR spectrum of TBDOPV

S4 Characterization of SP

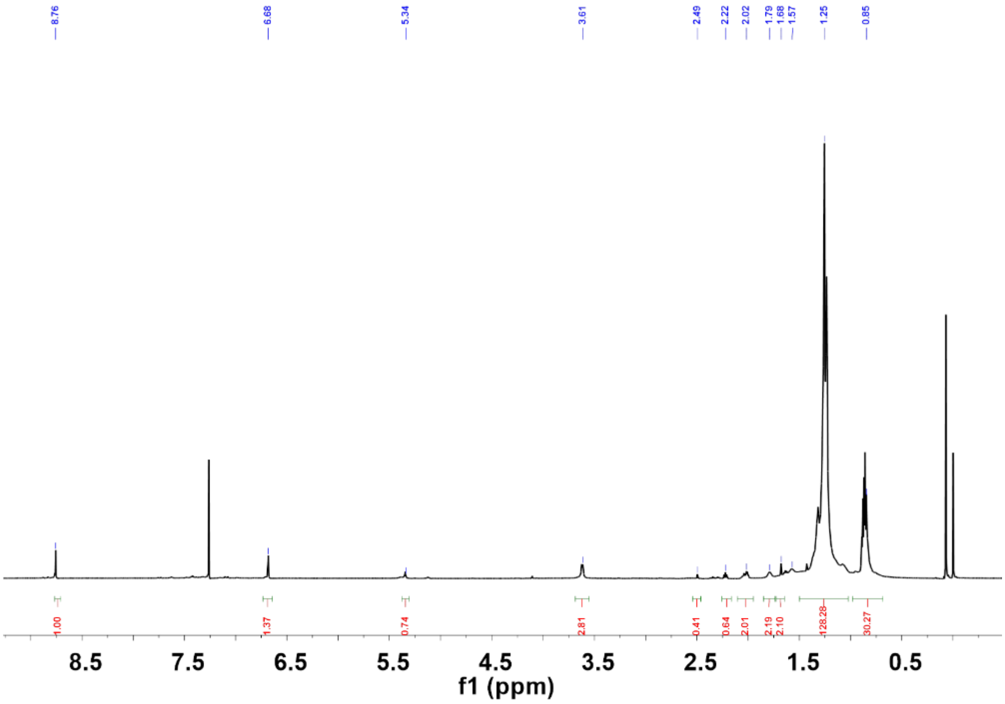

Fig. S4 ^{1}H NMR spectrum of SP

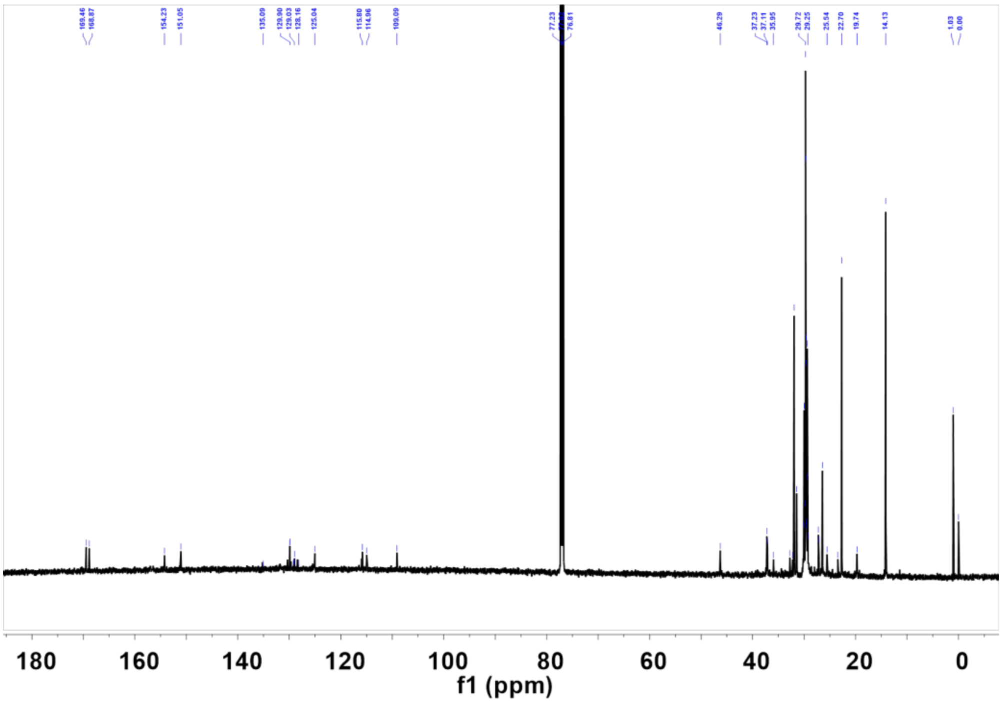

Fig. S5 ^{13}C NMR spectrum of SP

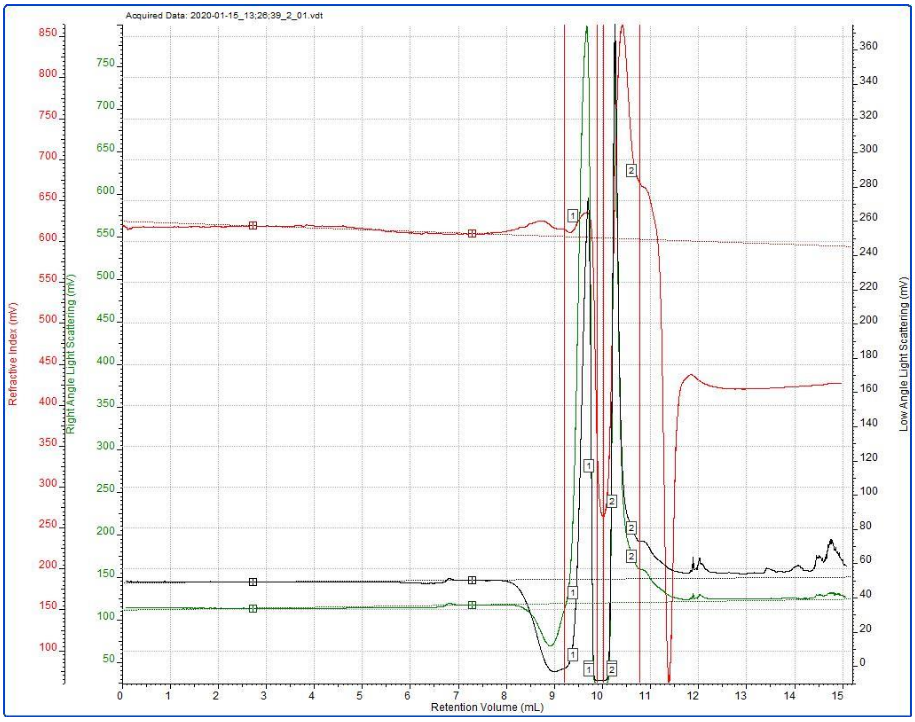

Fig. S6 GPC images of SP. The Mn of SP was determined to 6.366×10^6, with a polydispersity (Mw/Mn) of 1.668

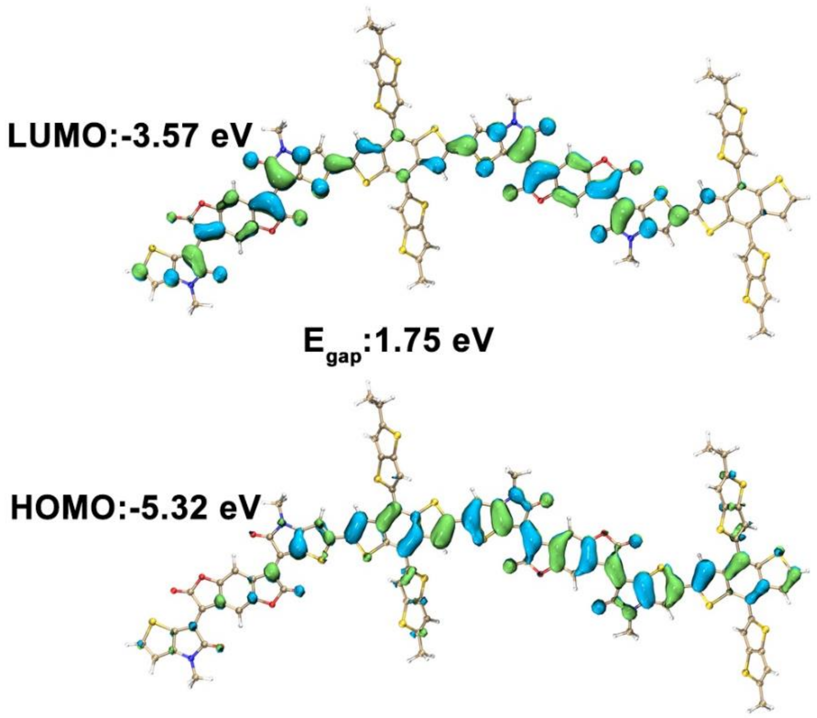

Fig. S7 Density functional theory (DFT) calculated HOMO and LUMO of SP dimer. DFT calculations were performed on the dimer to get insight on the frontier orbital distribution and level of the polymer. All optimizations were done at PBE0/def2-SVP level with Grimme's D3BJ empirical dispersion correction [S2]. The obital energies and distributions were obtained at same level of theory. All the calculations are performed using Gaussian09 program. $E_{gap} = E_{LUMO} - E_{HOMO}$

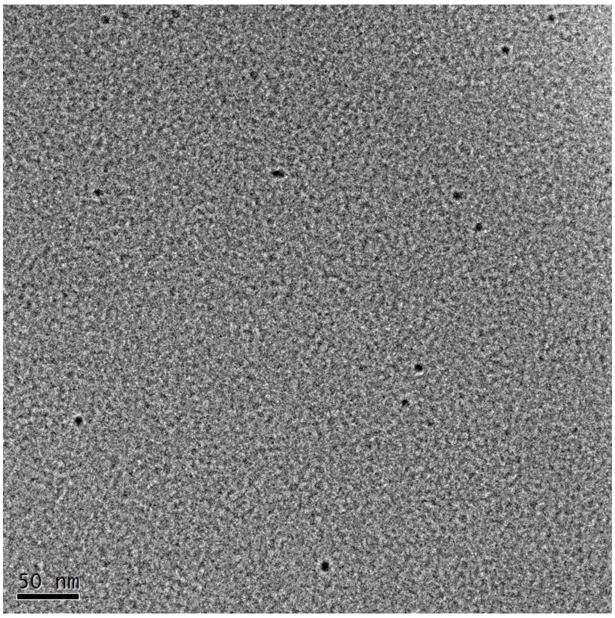

Fig. S8 TEM images of SPN@RBCM

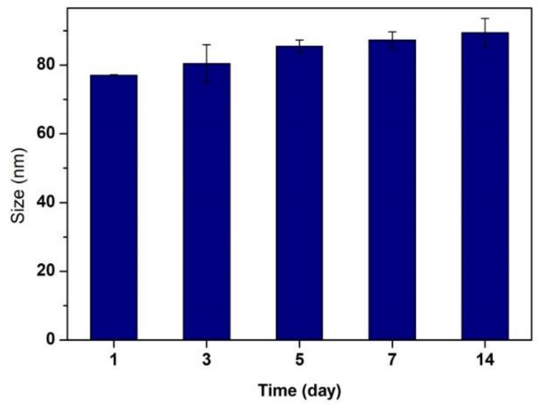

Fig. S9 Colloidal stability of SPN@RBCM in PBS with 10% FBS over a span of 14 days, as determined by DLS

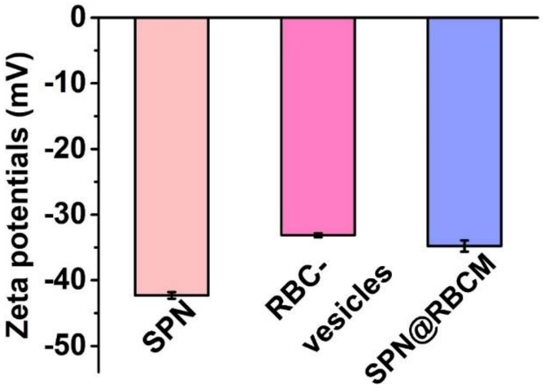

Fig. S10 Zeta potential of SPN, RBC-vesicles and SPN@RBCM

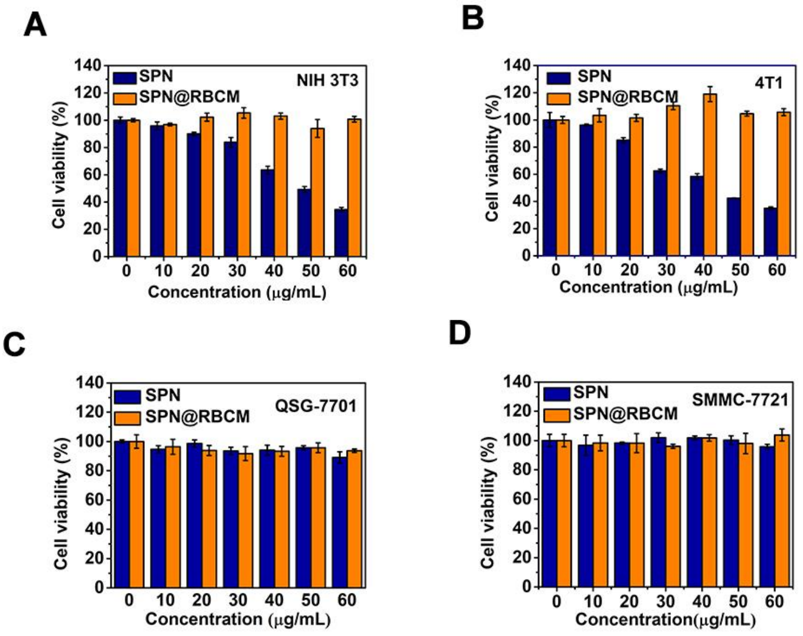

Fig. S11 Cell viability of (**A**) NIH-3T3, (**B**) 4T1, (**C**) QSG-7701, and (**D**) SMMC-7721 treated with different concentration of SPN or SPN@RBCM

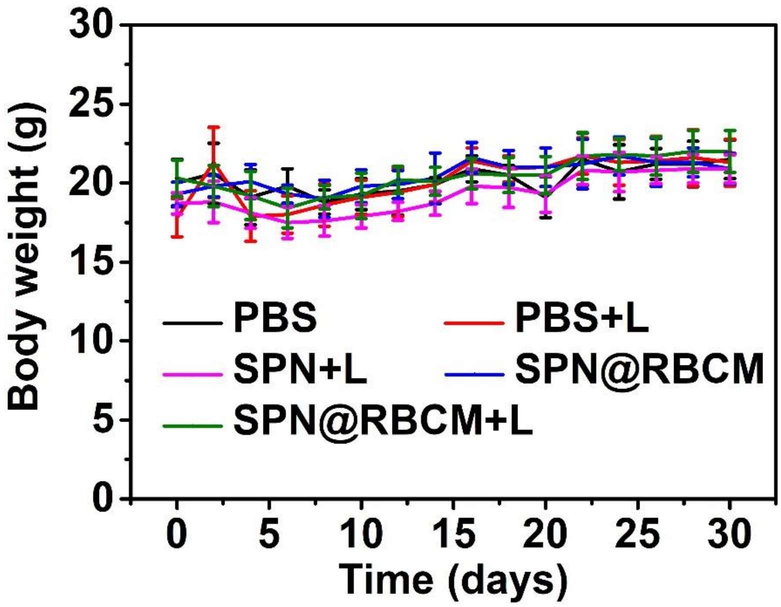

Fig. S12 Body weight variation profiles as a function of time after various treatments

Supplementary References

[S1]Y. Cao, J.-H. Dou, N.-j. Zhao, S. Zhang, Y.-Q. Zheng et al., Highly efficient NIR-II photothermal conversion based on an organic conjugated polymer. Chem. Mater. **29**, 718-725 (2017). https://doi.org/10.1021/acs.chemmater.6b04405

[S2]S. Grimme, S. Ehrlich, L. Goerigk, Effect of the damping function in dispersion corrected density functional theory. J. Comput. Chem. **32**, 1456-1465 (2011). https://doi.org/10.1002/jcc.21759
